# Fabrication of highly conductive graphene/ITO transparent bi-film through CVD and organic additives-free sol-gel techniques

**DOI:** 10.1038/s41598-017-18063-w

**Published:** 2017-12-19

**Authors:** Bastian Waduge Naveen Harindu Hemasiri, Jae-Kwan Kim, Ji-Myon Lee

**Affiliations:** Department of Printed Electronics Engineering, Sunchon National University, Suncheon, Jeonnam, 57922 South Korea

## Abstract

Indium tin oxide (ITO) still remains as the main candidate for high-performance optoelectronic devices, but there is a vital requirement in the development of sol-gel based synthesizing techniques with regards to green environment and higher conductivity. Graphene/ITO transparent bi-film was synthesized by a two-step process: 10 wt. % tin-doped ITO thin films were produced by an environmentally friendly aqueous sol-gel spin coating technique with economical salts of In(NO_3_)_3_.H_2_O and SnCl_4_, without using organic additives, on surface free energy enhanced (from 53.826 to 97.698 mJm^−2^) glass substrate by oxygen plasma treatment, which facilitated void-free continuous ITO film due to high surface wetting. The chemical vapor deposited monolayer graphene was transferred onto the synthesized ITO to enhance its electrical properties and it was capable of reducing sheet resistance over 12% while preserving the bi-film surface smoother. The ITO films contain the In_2_O_3_ phase only and exhibit the polycrystalline nature of cubic structure with 14.35 ± 0.5 nm crystallite size. The graphene/ITO bi-film exhibits reproducible optical transparency with 88.66% transmittance at 550 nm wavelength, and electrical conductivity with sheet resistance of 117 Ω/sq which is much lower than that of individual sol-gel derived ITO film.

## Introduction

Transparent conductive oxide (TCO) is one of the most important and extensively studied classes of advanced functional materials, with applications such as smart windows, flat panel displays, liquid crystal displays (LCD), organic light emitting diodes (OLED), solar cells, and touch screens^1–9^. Tin (Sn) doped indium oxide, also known as indium tin oxide (ITO), is still employed in TCO-based industry, and offers the best combination of high optical transmittance and low electrical resistance, which properties are sensitive to the synthesis methods^10–12^. The aqueous precursor sol-gel technique has recently become of interest compared with the other synthesis methods of ITO, such as rf and dc sputtering, spray pyrolysis, vacuum evaporation, and pulsed laser deposition, due to the simple and low synthesis cost for bulk-scale production, possibility to change the film microstructure, chemical stoichiometry, and easy introduction of a dopant^11–15^. However, the use of flammable and environmentally harmful organic solvent and additives as stabilizers and binders during the preparation process of organic based sol-gel is not suitable for mass industrial production^12,16^. Further, the aqueous sol-gel method, which uses water as the solvent, has become more convenient for mass production^17^. On the other hand, oxygen plasma treatment can be used to enhance the surface free energy (SFE) of the substrate, because of the wettability improvement of the substrate through the SFE enhancement^18,19^. This correlation between the SFE of the glass substrate and its wettability can be used to prepare a continuous high-quality thin ITO film on the glass substrate through the aqueous sol-gel spin coating technique. However, the lowest sheet resistance obtained through sol-gel spin coating technique is comparatively higher than that of ITO film produced by expensive methods, such as rf and dc sputtering and pulsed laser depositions, because the high porous structure and fine grain size of sol-gel derived ITO thin film is a significant disadvantage.

Graphene consists of a single layer carbon atoms arrangement in a hexagonal lattice, and its exceptional micromechanical and electron mobility properties, even at room temperature, make it a promising material for practical applications. Consequently it has received much attention in scientific studies^20,21^. This atomically thin two-dimensional material with an anomalous quantum Hall effect, massless Dirac electronic structure can be synthesized through exfoliation of graphite, epitaxial growth on electrically insulating surfaces, chemical reduction of graphite oxide, arc-discharge method, chemical vapor deposition (CVD) on a catalyst metal substrate, and so on^20,22^. Out of the above-mentioned methods, the CVD technique, since it was first reported in 2008, has become the most frequently used promising method for large-area high-quality graphene synthesis^17,20,23^. The graphene synthesis via CVD consists of chemical reaction of CH_4_, and deposition on a catalytical surface, such as Cu and Ni, at high temperature (1,000 °C)^20,24^.

Recently, it has been reported that hybrid bi-film, such as conductive metal oxide-carbon complexes, is an interesting new scientific field to achieve outstanding electrical and optical properties in an effective way^22,25,26^. The surface of this kind of combined materials shows lower sheet resistance than that of the individual surfaces, due to the improvement of surface carrier concentration in bi-film materials^22,25^. Further, high surface area semiconductive metal oxides, such as sol-gel derived ITO (high surface roughness promotes the high surface area), which does not have better electrical conductivity, can be used with graphene to improve the electrical performance more than that of individual sol-gel derived ITO, while keeping the optical transmittance at a desired level^27^.

Herein, the graphene/ITO conductive bi-film was synthesized to obtain 12.03% electical conductivity improvement of the sol-gel derived ITO via a two-step process: 10 wt.% tin-doped, ITO thin films were produced by an environment-friendly aqueous sol-gel spin coating technique on SFE enhanced glass substrate, and graphene was synthesized on Cu foil by a CVD method, and then transferred onto sol-gel derived ITO on glass substrate. The characteristics of sol-gel derived ITO, CVD graphene, the optical and electrical performances of graphene/ITO bi-film, and the effect of ITO on the Raman shift of CVD graphene will be addressed.

## Materials and Methods

### Synthesis of Organic Free ITO Sol-Gel

Indium(III) nitrate hydrate (In(NO)_3_.xH_2_O, 99.99% trace metals basis, Aldrich) and tin(IV) chloride (SnCl_4_, 99.995% trace metals basis, Aldrich) were used as initial materials for the synthesis of ITO. The hydration number of indium nitrate was determined by thermogravimetrical analysis (TGA), prior to mixing the materials. Indium tin solution was prepared by dissolving 1.938 g of In(NO)_3_.H_2_O and 0.324 g (145.55 µl) of SnCl_4_ in 15 ml of deionized water, and the resultant solution was refluxed at 40 °C for two hours. The concentration of Sn ion in the solution was adjusted to obtain Sn 10 wt. % for the final ITO film. The obtained sol was aged for 48 hours to obtain gelation, before going to the coating process.

### Preparation of ITO thin film

Square glass substrates of 20 × 20 mm size were used to deposit ITO by spin coating. The glass substrates were washed ultrasonically with acetone, isopropanol, and deionized water, respectively, and dried by using N_2_ gas flow. Prior to the spin coating, the cleaned glass substrates were oxygen plasma treated to improve the wettability and surface free energy of the surface, which helps to make continuous ITO film on the surface. The glass substrates were placed in the inductively coupled plasma (ICP) machine, and the chamber was vacuumed. The treatment was done by using oxygen gas with 85 sccm for 5 min under 150 W source power and 3.8 × 10^−2^ torr pressure. SFE was calculated by measuring the contact angles, and using the “Acid-Base (Van Oss-Chaudhury-Good)“ method.

1 $$\sqrt{{{\rm{\gamma }}}_{sv}^{LW}{{\rm{\gamma }}}_{lv}^{LW}}+\sqrt{{{\rm{\gamma }}}_{sv}^{acid}{{\rm{\gamma }}}_{lv}^{base}}=0.5\,{{\rm{\gamma }}}_{lv}(1+\,\cos \,{{\rm{\theta }}}_{y})$$

2 $${{\rm{\gamma }}}_{sv}^{total}={{\rm{\gamma }}}_{sv}^{LW}+2\sqrt{{{\rm{\gamma }}}_{sv}^{acid}{{\rm{\gamma }}}_{sv}^{base}}$$

where, ${{\rm{\gamma }}}_{sv}^{LW}\,\,$and ${{\rm{\gamma }}}_{lv}^{LW}$ are the Lifshitz-van der Waals components, and ${{\rm{\theta }}}_{y}$ is the contact angle between the solid and the measuring liquid.

From Equations (1) and (2), and the values in Table 1,

3 $${{\rm{\gamma }}}_{sv}^{LW}=12.7{(1+{\mathrm{Cos}{\rm{\theta }}}_{D})}^{2}$$

4 $${{\rm{\gamma }}}_{sv}^{acid}={(0.1747+6.0633{\mathrm{Cos}{\rm{\theta }}}_{F}-2.2757{\mathrm{Cos}{\rm{\theta }}}_{W}-3.6129{\mathrm{Cos}{\rm{\theta }}}_{D})}^{2}$$

5 $${{\rm{\gamma }}}_{sv}^{base}={(3.7385+9.4840{\mathrm{Cos}{\rm{\theta }}}_{F}-6.0633{\mathrm{Cos}{\rm{\theta }}}_{W}+0.3179{\mathrm{Cos}{\rm{\theta }}}_{D})}^{2}$$

${{\rm{\theta }}}_{D}$, ${{\rm{\theta }}}_{F}$, and ${{\rm{\theta }}}_{W}$ are the corresponding contact angles between the solid and Diiodomethane, between the solid and Formamide, and between the solid and deionized water, respectively.

**Table 1:** Surface tensions and components^67,68^.

Liquids	γ [mJm^−2^]	γ^*LW*^ [mJm^−2^]	γ^*acid*^ [mJm^−2^]	γ^*base*^ [mJm^−2^]
Deionized water	72.8	21.8	25.5	25.5
Formamide	58	39	2.28	39.6
Diiodomethane	50.8	50.8	0	0

A sufficient amount of prepared ITO gel was used to cover the glass substrate, and then it was spun at 3,000 rpm for 30 seconds. The coated substrates were dried at 100 °C for 15 min after each step. Spinning and drying cycles were repeated to get the desired thickness for the final film. Finally, coated substrates were annealed at 600 °C for 1 hour in low vacuum conditions about 0.15 Torr to complete the crystallization of ITO.

### Synthesis of Graphene & transferring

Graphene synthesis was performed under a CVD method by using over 99.99% pure and 25 µm thick Cu foil as the catalytic substrate. After being cut to 3 × 10 cm sizes, the Cu foil was treated with 10% diluted 48–51% HF at room temperature for 10 s to obtain Cu oxide free surface prior to graphene synthesis. The treated Cu foil was then placed in the CVD chamber, and heated up to 1,000 °C. 99.999% pure H_2_ gas was introduced into the chamber at 600 °C under 10 sccm flow rate, and the chamber pressure was maintained at 0.13 ± 0.01 Torr. At 1,000 °C, high quality pure CH_4_ gas was supplied into the chamber with 30 sccm flow rate with the continuous supply of H_2_ gas, and the chamber pressure was maintained at 0.5 ± 0.1 Torr. After 1 min time of CH_4_ gas supply, the supply of both gases, and the heater system were turned off, and the chamber system was cooled down to room temperature^28^.

The synthesized graphene was transferred onto ITO using a spin-coating of Poly(methyl methacrylate) (PMMA) - based indirect transfer method^29^. The PMMA (mol. wt. 22200, Aldrich) film was removed by using acetone and deionized water, respectively, and dried by using N_2_ gas flow. The graphene/ITO bi-films on glass substrates were then heated up to 100 °C for 30 min in normal atmosphere conditions. To compare the Raman signals of graphene on the synthesized ITO, a part of the prepared graphene were transferred onto SiO_2_ (300 ± 5 nm) /Si substrate, which was used as a reference substrate.

### Characterization

The crystal structure and crystalline phases of ITO thin films were analyzed by XRD with monochromatic Cu anode (K_α_ radiation with 1.54060 Å wavelength). The Scherrer equation was used to determine the crystallite size. Detailed information about the chemical composition of the ITO surface of the thin film was determined by XPS, and the elemental composition of the film was determined by EDX coupled with FE-SEM.

The quality of the synthesized graphene was characterized by Raman spectroscopy with 532 nm excitation laser beam, and data were collected at room temperature and normal atmosphere in the case of both SiO_2_/Si and ITO/Glass as substrates. The surface and cross-sectional (samples were cut in the middle of the ITO coated glass substrate) observation of both ITO thin film and graphene were done by using FE-SEM. The optical transmission and sheet resistance were measured by UV-Vis spectroscopy and four point probe head at room temperature, respectively. Electrical properties were also measured by using V-I characteristics.

## Results and Discussion

### SFE dependent surface morphology of ITO for the bi-film

**Figure 1:**
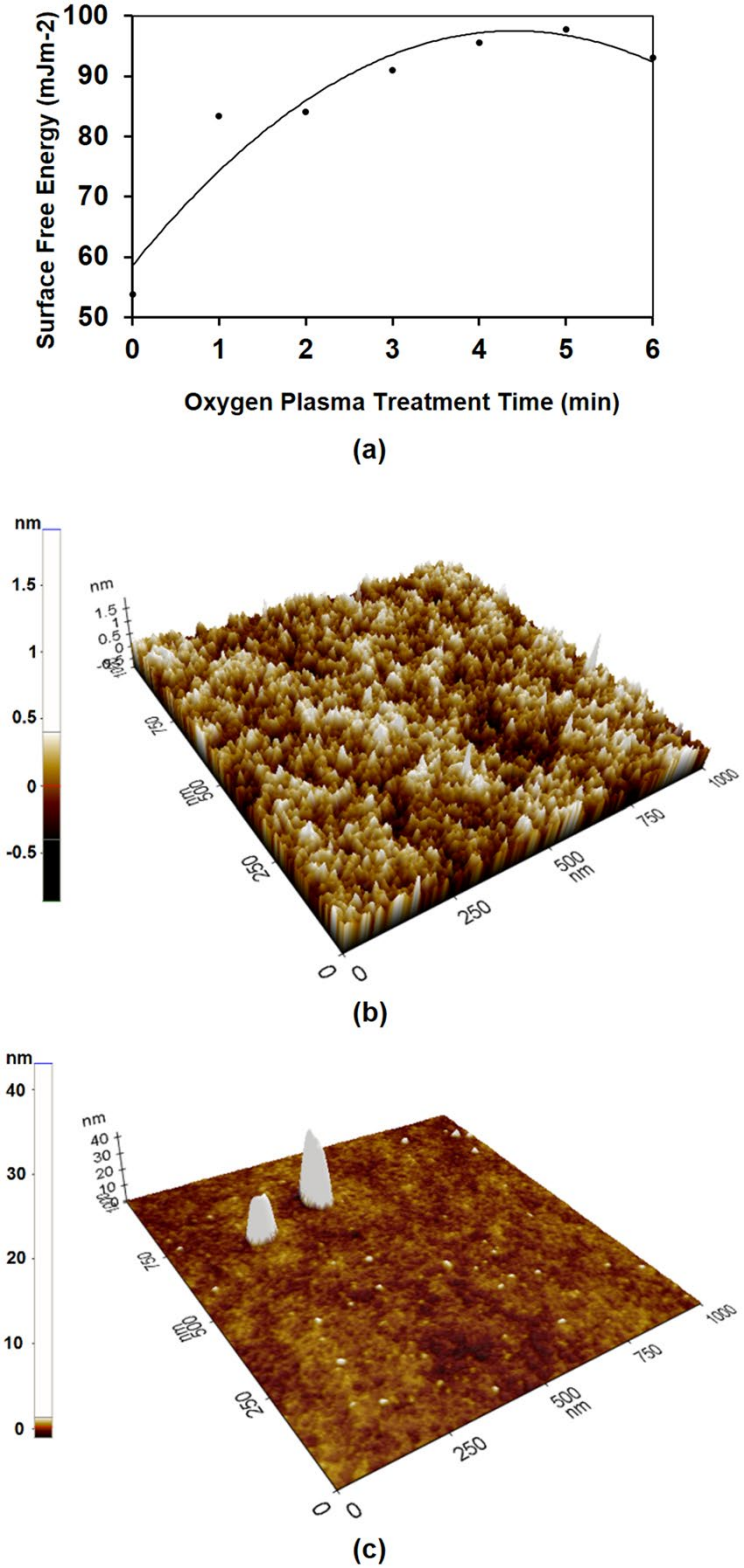
(**a**) Surface free energy of glass substrate with oxygen plasma treatment time. AFM (atomic force microscopy) scan of glass substrates: (**b**) without oxygen plasma treatment, and (**c**) with 5 min of oxygen plasma treatment.

Figure 1(a) shows the SFE variation of the glass substrate with oxygen plasma treatment time. Initially, the SFE associated with chemically cleaned glass (without oxygen plasma treatment) was 53.826 mJm^−2^. The SFE increased with the oxygen plasma treatment time, and it reached a maximum value of 97.698 mJm^−2^ after 5 min. Figure 1(b,c) show the AFM scan of chemically cleaned glass substrates, without oxygen plasma treatment, and with 5 min of oxygen plasma treatment, respectively. The oxygen plasma treatment introduces micro roughness of the glass surface through the surface damage and fine irregularities on the surface, which improves the wettability of the glass surface^30,31^. In fact, at 5 min of oxygen plasma treatment, the glass was severely damaged, causing a high surface root mean squared (RMS) roughness of about 213 pm, with respect to the oxygen plasma un-treated glass with a surface RMS roughness of about 172 pm. Moreover, slight SFE reduction of the glass surface with further oxygen plasma treatment was also observed, and after 6 min, it reduced to 93.012 mJm^−2^. This SFE reduction may occur during continuous oxygen plasma treatment (after 5 min of time), due to surface smoothness through the initiation of the splitting of hills, which occurred at the initial stage of oxygen plasma treatment of the glass substrate^30^.

**Figure 2:**
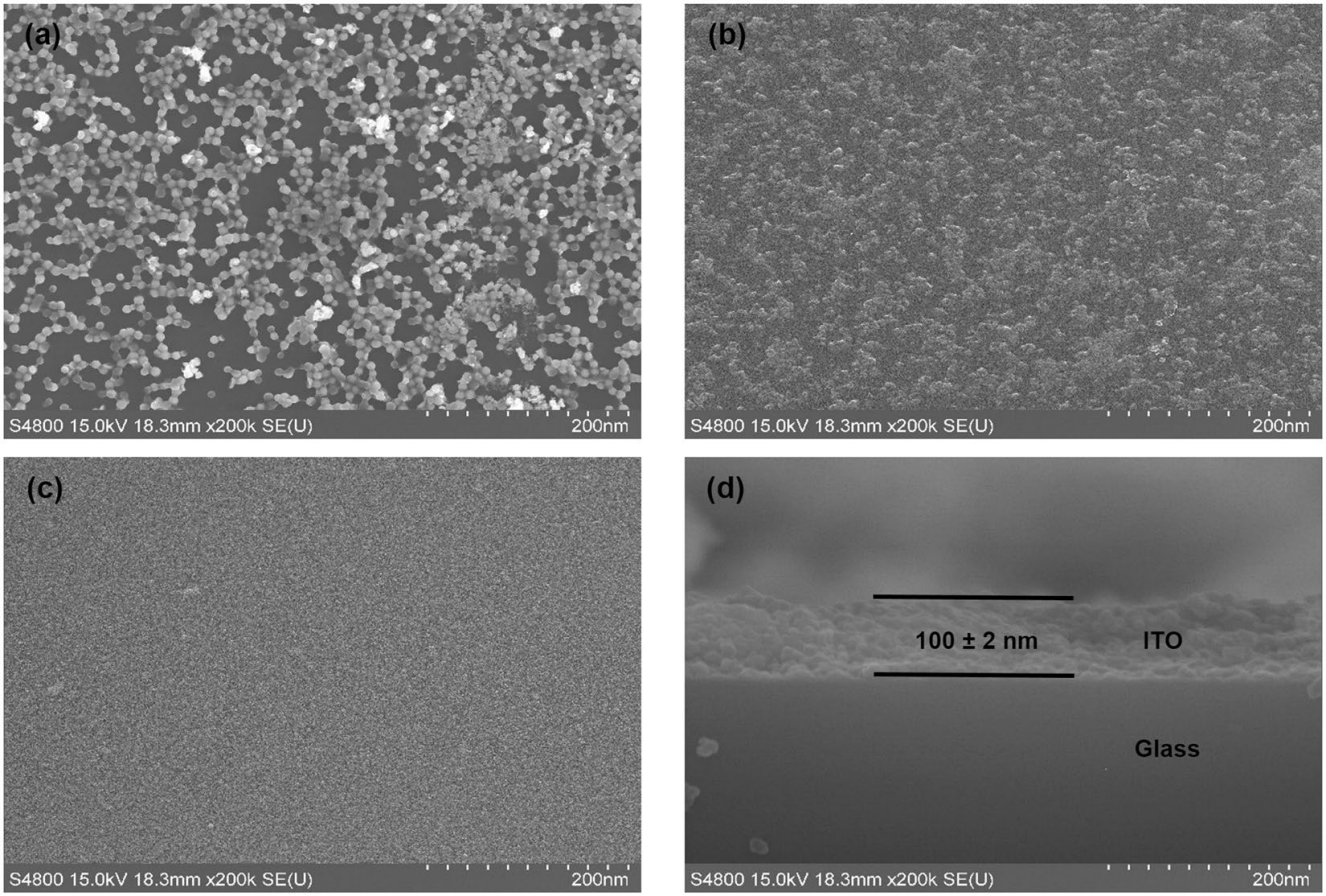
FE-SEM imagery of ITO coated glass substrate. (**a**) 1 time ITO coated on chemically cleaned (without oxygen plasma treatment) glass substrate, (**b**) 6 times ITO coated on chemically cleaned (without oxygen plasma treatment) glass substrate, (**c**) 6 times ITO coated on oxygen plasma treated (5 min) glass substrate, and (**d**) cross-sectional FE-SEM image of 6 times ITO coated on oxygen plasma treated (5 min) glass substrate.

Figure 2(a) shows the FE-SEM imagery of one single spin coated ITO after annealing at 600 °C for 1 hour on chemically cleaned glass substrate (oxygen plasma untreated). It was clearly observed that the chemically cleaned glass with low SFE (53.826 mJm^−2^) produced discontinuous, highly dispersed, agglomerated ITO islands, instead of continuous layer. Continuous ITO film was not obtained, even after a number of multiple coatings of ITO sol-gel on oxygen plasma untreated glass substrate, and Fig. 2(b) shows that particles were highly agglomerated, and made irregular shape clusters. The reason for this discontinuous structure is the low wetting properties of glass surface during spin coating of ITO gel. Figure 2(c) shows that the SFE enhanced glass substrates through 5 min of oxygen plasma treatment produced smooth and void-free continuous ITO film on the glass surface, because of the wettability improvement through the oxygen plasma treatment of the glass surface, which facilitates high adhesion of ITO gel to the glass surface. Figure 2(d) shows cross-sectional FE-SEM imagery of 6 times spin coated ITO on 5 min oxygen plasma treated glass substrate. Moreover, a homogeneous microstructure was observed, consisting of spherical shape with different sizes of grains. Further, the grain boundaries of the ITO film were well defined. The thickness of the ITO film after 6 times spin coating as measured by alpha step equipment was 100 ± 2 nm; and it showed the lowest sheet resistance of 133 Ω/sq according to the four point probe method, so it was used to fabricate the graphene/ITO bi-film.

### Crystallinity of the ITO thin film

**Figure 3:**
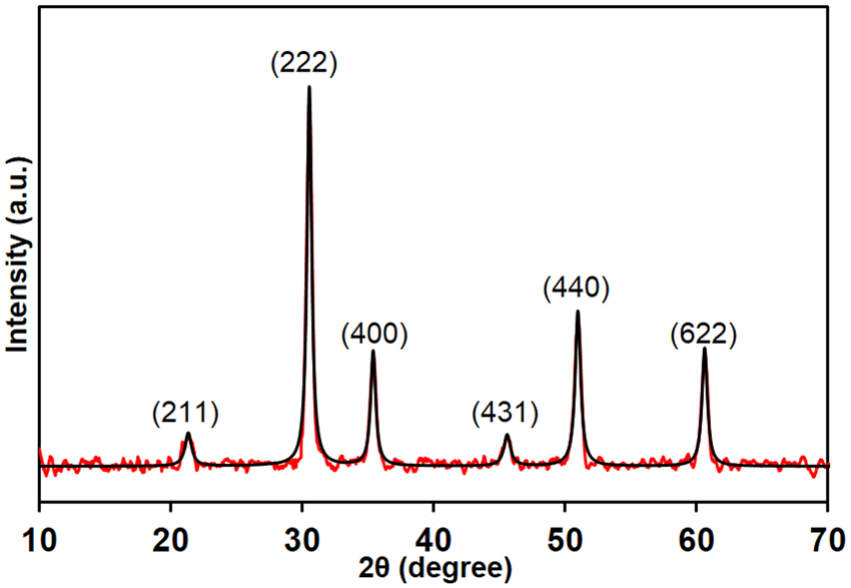
X-ray diffraction pattern of ITO film (red line) and Lorentzian fitted curve (black line) after annealed at 600 °C for 1 hr in vacuumed conditions.

Figure 3 shows the crystal phases of the ITO film on oxygen plasma treated glass substrate (annealed at 600 °C for 1 hour), which were obtained by X-ray diffraction (XRD) pattern and Lorentzian fitting curve, and reveals the most intense peaks of (211), (222), (400), (431), (440) and (622). The sharp peak with high intensity corresponds to the (222) plane, which is identical to the (111) preferred orientation of the ITO film. This strong (222) peak reveals that the preferred orientation of crystal growth of the ITO is in the < 111 > direction. During annealing at high temperature, the amorphous gel was decomposed, and transformed to cubic polycrystalline structure with (111) preferred orientation. The pure In_2_O_3_ has [100] direction orientation, and obtained (111) orientation indicates that doped tin substitutionally replaces indium in the lattice structure^3,13,14,32^. The peaks of (211), (222), (400), (431), (440) and (622) were found at 21.13°, 30.52°, 35.42°, 45.73°, 50.95° and 60.63°, respectively. The absence of characteristic peaks at 26.5° and 33.2°, which correspond to SnO_2_ and SnO respectively, reveals that one single phase of In_2_O_3_ is present, without any single phase of Sn in the ITO film. The interplanar spacing of (222) was obtained as 2.926 Å, which shows good agreement with 2.921  Å, which is the interplanar spacing of (222) according to the joint committee on powder diffraction standards (JCPDS) card. The obtained crystal quality parameter I_222_/I_400_ (intensity ratio of (222) and (400)) was 3.301. Furthermore, the crystallite sizes of ITO were determined from the line broadening nature of the XRD pattern according to the Scherrer formula^12,32,33^, and the obtained crystallized sizes for (222) and (400) peaks were 14.35 ± 0.5 and 14.37 ± 0.5 nm respectively (the corresponding full-width at half-maxima, FWHM were 0.5672° and 0.5734°, while the corresponding Bragg angles were 15.26° and 17.71°, respectively).

### Chemical and elemental composition of the ITO Thin Film

**Figure 4:**
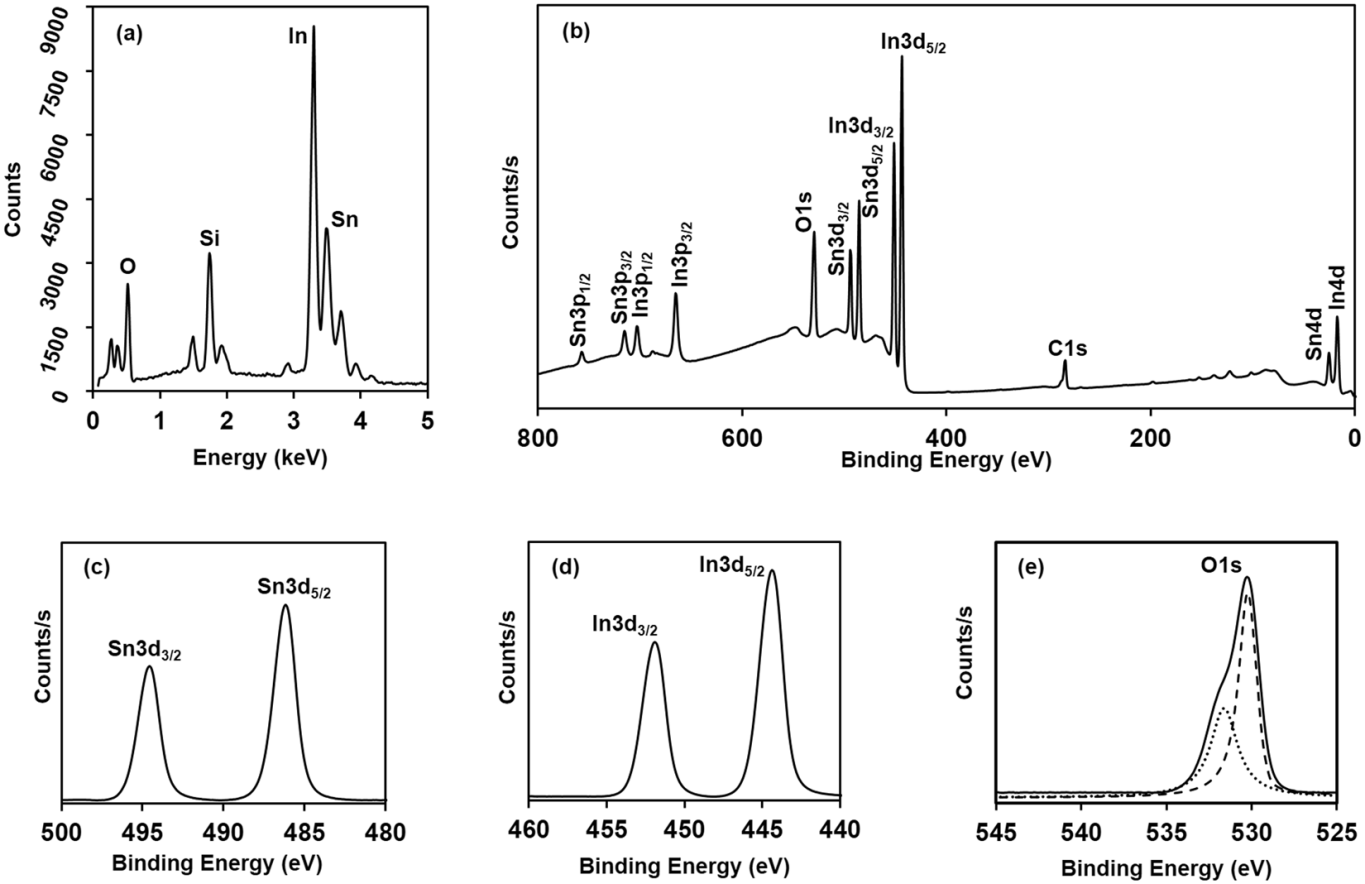
(**a**) Energy dispersive X-ray spectroscopy of ITO film (annealed at 600 °C for 1 hr in low vacuumed conditions), and (**b**)–(**e**) X-ray photoelectron spectroscopy of ITO film (annealed at 600 °C for 1 hr in low vacuumed conditions): (**b**) Wide scan spectra, (**c**) narrow scan spectra of Sn3d doublet, (**d**) In3d doublet, and (**e**) O1s (dotted and discontinuous line graphs show two fitted curves at a binding energy of 530.21 and 531.63 eV).

It is very important to maintain the Sn doping value within a certain level limit to obtain better electrical properties of ITO film, and previously reported research found that 10 wt. % Sn content ITO shows the minimum sheet resistance^4,11,13^. When the content of Sn is increasing, all the Sn atoms cannot participate in substitution for In atom, and Sn ions closely pack to each other, which makes a defect complex with average electrical charge of + 3, due to the tendency of Sn(IV) conversion to Sn(II). These Sn ions in the defect complex do not participate in the electrical conductivity^6^. The bulk chemical composition present in the ITO film was determined by energy dispersive X-ray spectrometry (EDX) along with FE-SEM, as shown in Fig. 4(a). The results show that around 10 wt. % of Sn entered into the In_2_O_3_ lattice. A peak relevant to the Si was obtained in EDX, due to the use of glass as a substrate for ITO coating.

Figure 4(b) shows X-ray photoelectron spectroscopy (XPS) survey scan data of the elements present in the ITO film. Figure 4(c–e) show the XPS narrow scan spectra in the range of Sn3d, In3d and O1s, respectively. The C peak is also present, even though In, Sn and O are the expected chemical elements in the ITO film. The main reason for this C peak is contamination of the ITO surface during sample preparation^34^. The peak positions of In3d5_/2_ and Sn3d_5/2_ corresponding to the metallic In and Sn occur at 443.6 ± 0.2 and 484.5 ± 0.2 eV, respectively^35^. The peak positions of In3d_5/2_ and Sn3d_5/2_ for the prepared ITO film are around 444.28 and 486.7 eV, respectively. The chemical shifts of In3d_5/2_ and Sn3d_5/2_ from metallic form to the prepared ITO film were 0.68 ± 0.2 and 2.2 ± 0.2 eV, respectively, which clearly reveals the prepared ITO has only the oxide forms of In and Sn, and is free of metallic compounds.

On the other hand, to know the oxidation state of Sn is very important, because the electrical conductivity reduces with reduction of Sn^4+^ to Sn^2+^ ^34^. The peak positions of Sn^4+^3d_5/2_ and Sn^2+^3d_5/2_ would be at 486.8 and 485.6 eV, respectively, and the 1.2 eV peak shift would be associated with the reduction of Sn^4+^ ^34^. However, no peak at around 485.6 eV was observed, which revealed that during the preparation of ITO sol-gel and the annealing process of ITO film, there was no Sn^4+^ reduction to Sn^2+^. Figure 4(e) shows the fitting of the O1s spectra (dotted and discontinuous curves) with two peaks located at binding energies of 530.21 and 531.63 eV, respectively; both peaks are possible in crystalline ITO, due to two different possible locations for the O^2-^ lattice ions in In_2_O_3_, namely the oxygen-deficient region, and non-deficient region^6^.

### Raman characteristic features and surface morphology of CVD graphene in bi-film

**Figure 5:**
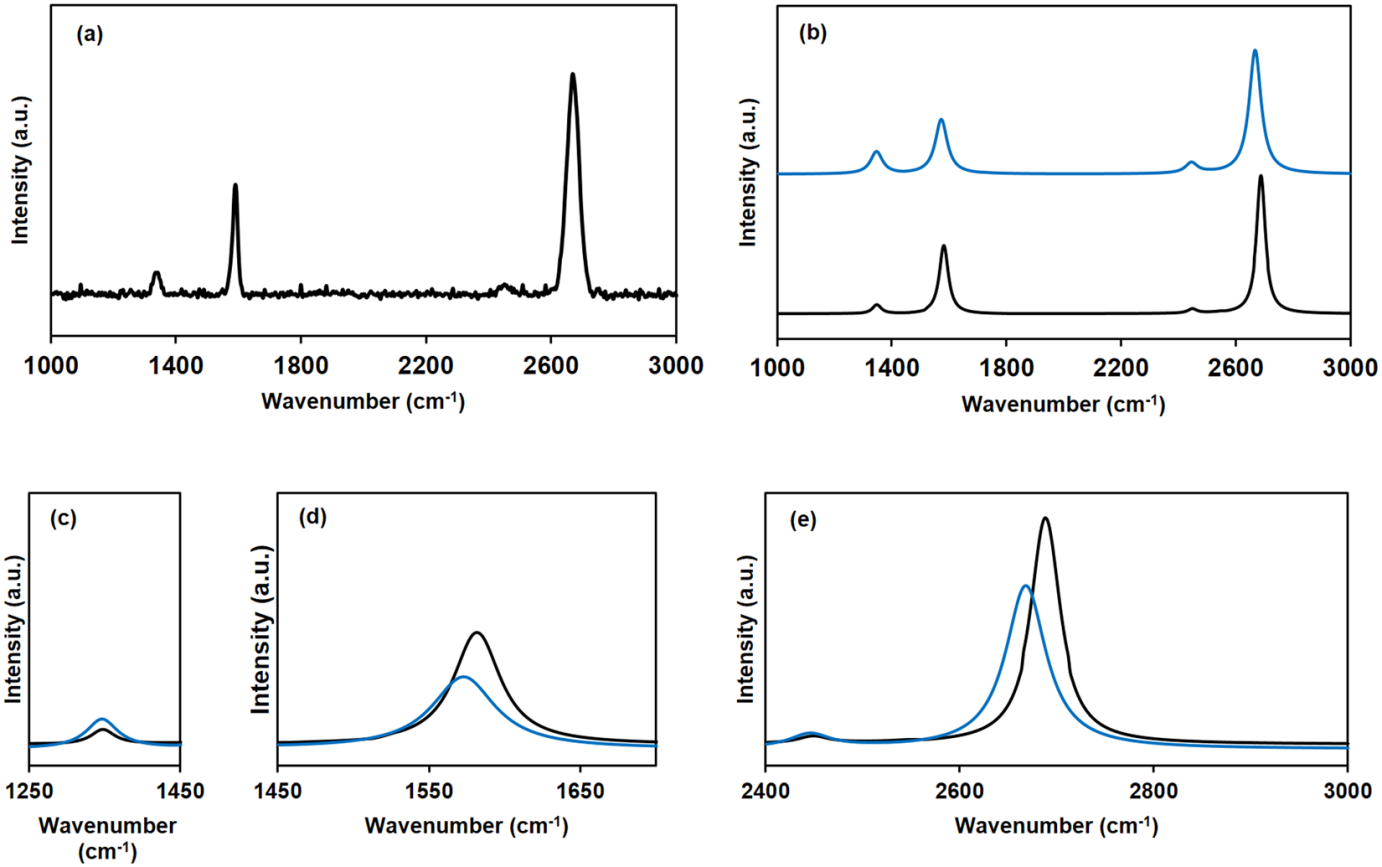
(**a**) Raman spectrum of CVD graphene (excitation wavelength 532 nm) after transference to SiO_2_ (300 ± 5 nm)/Si, (**b**) Lorentzian fitted graphs [blue graph corresponds to graphene on ITO (100 ± 2 nm)/glass, black graph corresponds to graphene on SiO_2_ (300 ± 5 nm)/Si], and zoomed spectra of the (**c**) D peak, (**d**) G peak, and (**e**) 2D peak.

Raman spectroscopy was used to characterize the synthesized graphene because of its non-destructive nature, and the possibility of providing not only a wide variety of information, such as disorder, the number of layers, grain boundaries, and doping, but also the mechanical, electrical and optical properties^20,21,36^. Fig. 5(a) represents the corresponding Raman graph for 532 nm excitation of synthesized graphene on SiO_2_ (300 ± 5 nm)/Si substrate (here, SiO_2_ (300 ± 5 nm)/Si was used as a reference substrate), the G and 2D bands of synthesized graphene on SiO_2_/Si appeared at 1,582.58 and 2,688.69 cm^−1^ respectively, with a very low intense D peak at 1,348.35 cm^−1^. The FWHM of the 2D band was around 28 cm^−1^, which can be used as a quantitative analysis of the number of layers of graphene, and the FWHM of G band was around 19 cm^−1^. The determined *I*_*2D*_ */I*_*G*_ (intensity ratio of 2D to G) and *I*_*D*_ */I*_*G*_ (intensity ratio of D to G) of graphene on SiO_2_/Si were 2.11 and 0.098, respectively. Figure 5(b) shows the Lorentzian fitting curves of synthesized CVD graphene, where the black graph represents the graphene on SiO_2_/Si, and the blue graph represents the graphene on ITO/glass. These Lorentzian curves can be used along with the *I*_*2D*_ */I*_*G*_ to represent the number of layers in graphene^21,37–40^. The single Lorentzian feature of the 2D band at room temperature, as shown in Fig. 5(b), and 2.11 of *I*_*2D*_ */I*_*G*_ indicate the existence of monolayer graphene, since at room temperature, the 2D band for monolayer graphene shows a single Lorentzian feature, while bilayer graphene shows four Lorentzian features. The same single Lorentzian feature was also observed after graphene was transferred to the ITO/glass; however, in contrast, the G and 2D bands were broader, and a comparatively high D peak was observed, as shown in the blue graph of Fig. 5(b). Further, the above-mentioned D, G, and 2D peaks appear at 1,346.34, 1,573.63, and 2,666.29 cm^−1^, respectively, when the CVD graphene was transferred to the ITO spin-coated glass. In other words, on average, the positions of D, G, and 2D peaks were red shifted by 2, 9, and 22 cm^−1^, respectively. Figure 5(c–e) show the zooming Raman spectra around the D, G and 2D peaks in the same dimensions, respectively (the blue graph corresponds to graphene on ITO/glass, while the black graph corresponds to graphene on SiO_2_/Si).

**Figure 6:**
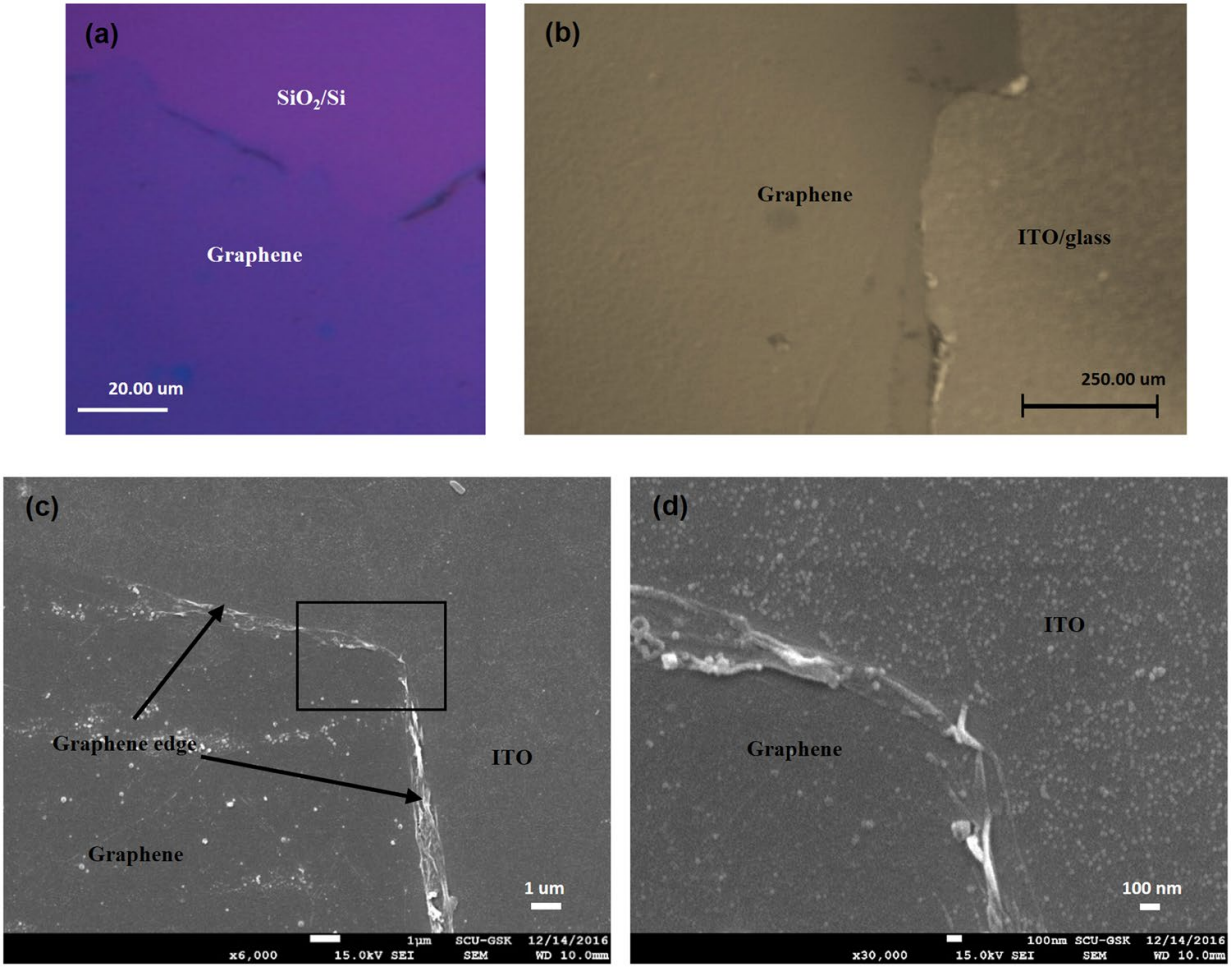
Optical microscopic imagery of (**a**) graphene on SiO_2_ (300 ± 5 nm) /Si substrate, and (**b**) graphene/ITO bi-film (graphene on sol-gel derived ITO (100 ± 2 nm)/glass substrate). FE-SEM imagery of graphene/ITO bi-film, (**c**) at × 6,000 magnification, and (**d**) at × 20,000 magnification, of area near the graphene edge.

The peak positions depend on not only the material structure, but also the external factors, such as doping level, stress/strain conditions, temperature, and environmental effects; the substrate-induced charge transfer by n-type doping affects red-shift, while p-type doping affects the blue shift of both 2D and G bands^41–44^. Moreover, it was also known that when the graphene is in strain conditions due to the elongation of carbon – carbon bond, the breaking of symmetry and anisotropy nature of the graphene would strongly affect the redshift of Raman peaks^45,46^. The stress/strain might be initiated due to the lack of surface smoothness of the sol-gel derived ITO (n-type material) coated glass. The heating effect during Raman spectra analysis may not have affected the shift of G peak position, since data was collected at room temperature, and all the observed Raman red-shifts were more than 2 cm^−1^, which is the previously reported red shift due to the heating effect^47,48^. In addition, the G peak position is known to not be changed, but would be split when the glass surface affects the Raman phenomena of the graphene^49–50^. However, that kind of glass surface-induced splitting of G peak was not observed in the above Raman spectrum, even after the graphene transference onto the ITO coated glass substrate. According to the obtained results, the most interesting fact is that this red shift highly influenced the 2D band more than the G band, and the red shift of the 2D peak is around 2.4 times higher than that of the G peak. Further, the D peak of graphene on ITO/glass was intense, while both G and 2D peaks were reduced, when compared with those peaks in the case of graphene on SiO_2_/Si. The *I*_*D*_ */I*_*G*_ of graphene on the ITO/glass was 0.392, and it represents that the concentration of defects is higher than that of graphene on SiO_2_/Si (*I*_*D*_ */I*_*G*_ of graphene on SiO_2_/Si was 0.147). This defective nature occurs during the transference of graphene onto the rough surface of synthesized ITO. The graphene transfer process tends to form wrinkles in graphene; moreover, the high surface adhesion in rough substrate helps to increase the number of wrinkles^51^. Fig. 6(a,b) show optical microscopic imagery of the synthesized CVD graphene on SiO_2_/Si substrate and graphene/ITO bi-film, respectively. The continuous graphene layer can be easily observed on the SiO_2_/Si substrate. However, while it is difficult to use the optical microscope to observe the continuity of CVD graphene on ITO in bi-film, even the graphene edges can be easily identified on the ITO. Figure 6(c,d) show the FE-SEM images of graphene/ITO bi-film on the glass substrate in different magnification at the edge of graphene, which confirms the continuous existence of CVD graphene on the wide area of sol-gel derived ITO. The zoomed image of the square region in Fig. 6(c) is shown in Fig. 6(d), which shows wrinkles of the graphene edge.

### The optical and electrical performances of the graphene/ITO bi-film

**Figure 7:**
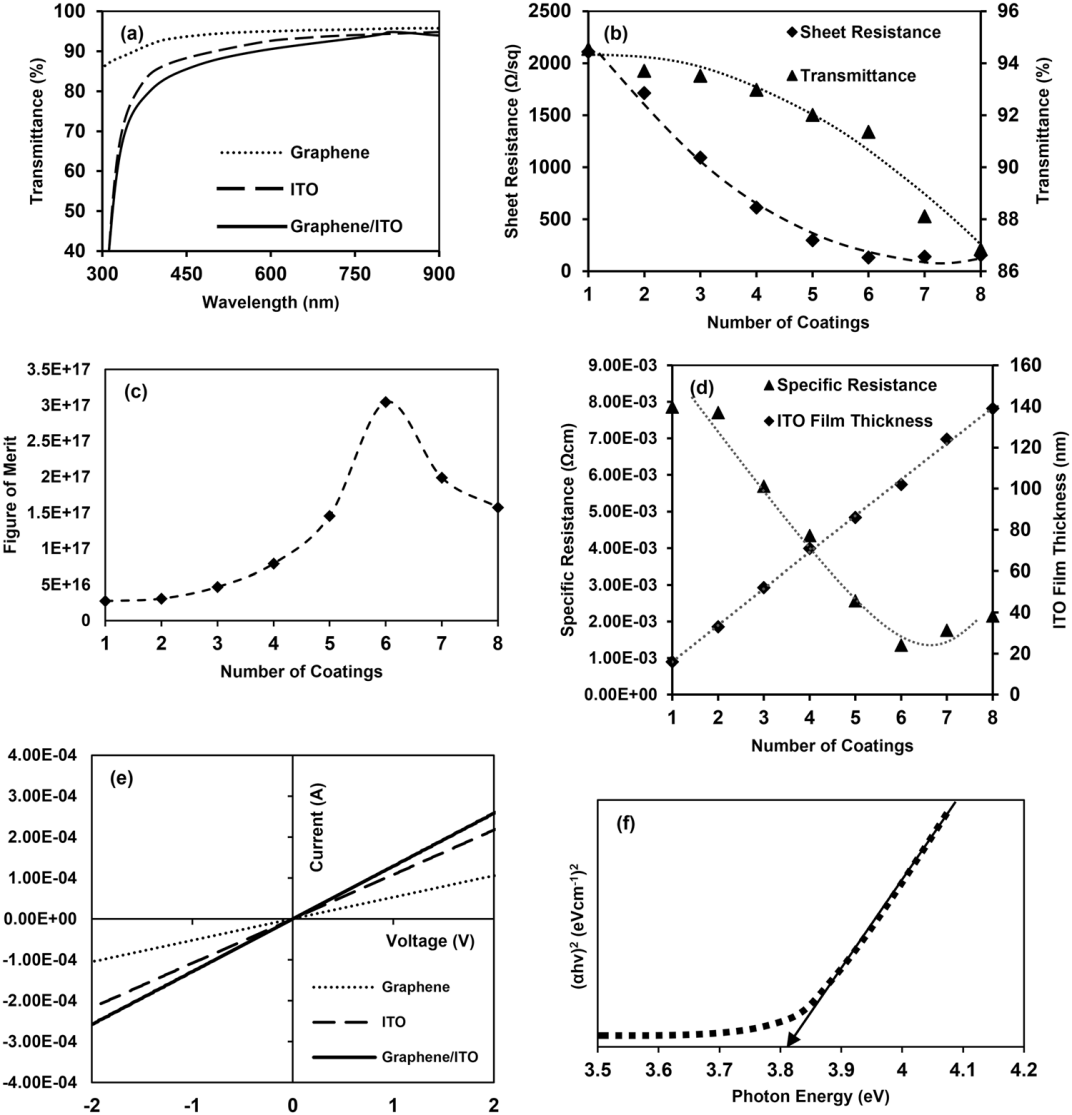
(**a**) UV-Vis spectrum of graphene, ITO film, and graphene/ITO bi-film (baseline was corrected by using 5 min oxygen plasma treated glass substrate), (**b**) sheet resistance (Ω/sq) and transmittance (%) at 550 nm wavelength depending on the number of ITO coatings, (**c**) figure of merit (T^10^/R_s_, where T is the optical transmission at 550 nm wavelength and R_s_ is the electrical sheet resistance) of the ITO film depending on the number of ITO coatings, (**d**) specific resistance (Ωcm) and thickness of the ITO film depending on the number of ITO coatings, (**e**) V-I characteristics of graphene, ITO film and graphene/ITO bi-film, and (**f**) the (αhν)^2^ versus hν plot of graphene/ITO bi-film.

Figure 7(a) shows the light transmittance of sol-gel derived ITO (100 ± 2 nm thickness), synthesized CVD graphene, and fabricated graphene/ITO (100 ± 2 nm thick ITO film) bi-film. The synthesized graphene shows 96.94% optical transmittance at 550 nm wavelength. In comparison with the other two, the transmittance of the bi-film is a little bit lower in the 300–900 nm wavelength range, due to light absorption in both ITO film and graphene during the light transmission. The fabricated bi-film shows 88.66% transmittance at 550 nm wavelength, and the reductions of transmittance at the same wavelengths were 2.95% and 8.54%, respectively, when compared with 100 ± 2 nm thick ITO film and CVD graphene. Over 595 nm wavelength, the bi-film shows more than 90% transmittance. This indicates that the light transmittance of the bi-film is strongly dependent on the number of coatings of the ITO sol-gel during the preparation of ITO film, due to the unique optical properties of 2-dimensional graphene. Figure 7(b) shows that the transmittance of ITO only-film reduced with the increased number of coatings, due to the inversely proportional relationship of transmittance with the thickness of the substances.

Figure 7(c) shows the figure of merit of ITO (F_ITO_) film according to the number of coating times, which was calculated by the following equation, where T is the optical transmission at 550 nm wavelength, and R_s_ is the electrical sheet resistance^52^.

6 $${{\rm{F}}}_{{\rm{ITO}}}=\frac{{T}^{10}}{{R}_{s}}$$

The highest F_ITO_ was obtained from six times coated ITO film, indicating that the transmittance of the bi-film is highly dependent on the properties of ITO film.

Simultaneously, the sheet resistance of the ITO film was also reduced up to six times of coating, with film thickness and sheet resistance of 100 ± 2 nm and 133 Ω/sq, respectively. As with the light transmittance, the sheet resistance of the bi-film is also dependent on the thickness of the ITO film that was used to produce the bi-film. After six times of ITO coating, a slight increment of sheet resistance was also observed. This behavior occurs in the sheet resistance of the ITO film due to the initiation and propagation of defects, the increment of micro-cracks, agglomeration of materials, and lower interaction within each layer and with the surface, after a certain thickness of the ITO film^16^. The thickness of the ITO film for 6 times coating was 100 ± 2 nm, which indicates that the thickness of one single coating of ITO is approximately 16.5 nm. The sheet resistance of the synthesized graphene was around 700 Ω/sq. The most interesting observation is that the sheet resistance of bi-film was 117 Ω/sq, which is much better electrical performance improvement of ITO (12.03%) compared to the previously reported value (2.39%) of graphene on ITO film by Liu J. *et al*. The improvement of electrical conductivity of the sol-gel derived ITO after the introduction of graphene on it occurs due to carrier mobility and surface carrier concentration improvement through graphene. Since the conductivity is proportional to both the carrier concentration and the carrier mobility, the same conductivity could be achieved from a large value of carrier concentration with a small value of carrier mobility, or a small value of carrier concentration with a high value of carrier mobility^53^. Graphene has high carrier mobility and of higher value than that in ITO^22^. However, even though the carrier density of graphene is lower than in ITO, it could be improved through the introduction of n/p doping from a foreign material, such as ITO in the present study. Therefore, by using graphene in contact with ITO, both the carrier concentration and carrier mobility of the bi-film could increase more than the values of the individual ITO. Furthermore, although the intrinsic carrier mobility of graphene would be degraded, due to scattering caused by charged impurities located near graphene/ITO interfaces, and contact resistance between graphene and ITO, the value remains higher than in individual ITO^22^. As both the carrier concentration and carrier mobility in bi-film show more increased values than those values in individual ITO, the bi-film shows better conductivity or current than does individual ITO. Moreover, graphene provides surface smoothness to the bi-film, which helps to enhance the electrical performances of high surface rough sol-gel derived ITO^22^. Fig. 7(d) shows the behavior of the specific resistance and thickness of the ITO film as a function of the number of coatings. The specific resistance of the ITO film behaved similarly to the sheet resistance of the ITO film with the number of coatings, as mentioned above.

The existence of electron scattering due to surface roughness of conductive films has been known for a lengthy time ago and was formulated and showed by Thompson and later in more details by Fuchs^54,55^. Additional resistivity is induced due to diffuse scattering of conduction electrons on the rough surface, and effect of surface roughness on electron scattering increases with increase of surface roughness. Conduction electrons impact each other during transferring and this acts as a barrier for free electron movement due to the effect of surface roughness scattering^57^. Effect of surface roughness on electron transfer can be explained in the following ways. Surface roughness introduces a variation of film thickness, causing fluctuations in electrostatic potential of the film and thus in the electron sub-band profile. Surface roughness changes the thickness of the film, introducing external variations in the sub-band energy, as well as fluctuations in the wave function shape^56^. Moreover, the Born approximations can be used to express the conductivity of film when surface roughness contributes to electron scattering^57^. In the framework of this theory, surface roughness scattering is becoming more critical due to rapid variation of film thickness and resulting in reduction of electrical conductivity^55^. Electron scattering can be reduced through improvement of surface smoothness, resulting in improvement of electrical properties through improvement of electron mobility. The conductivity of sol-gel derived ITO with rough surface can be improved by introducing graphene on ITO, since graphene acts as a bridge on the rough ITO surface, which facilitates a smooth path for electron transfer. In addition to the surface smoothness improvement of ITO from graphene, the carrier mobility of bi-film is improved over that of individual ITO^22^. So the surface carrier density improvement through electron doping from ITO and the carrier mobility improvement from graphene facilitate higher conductivity or lower sheet resistance to bi-film, when compared with the individual ITO. ITO film obtained from this study reveals high surface root mean squared (RMS) roughness of about 14.398 nm and mean roughness of 12.522 nm that comparatively much higher than reported roughness values of ITO from other synthesis method such as radiofrequency (RF) magnetron sputtering^57–59^. Graphene/ITO bi-film with surface RMS roughness of approximately 9.387 nm and mean roughness of 7.322 nm, indicating that surface roughness of ITO film can reduce by coating graphene on it (see Supplementary Fig. S1). Results obtained from the Hall Effect system suggest that graphene improves surface carrier concentration and carrier mobility of bi-film relative to individual ITO synthesized by sol-gel method (see Supplementary Table S1). The lowest sheet resistance obtained from the sol-gel method is comparatively higher than that of other expensive methods, such as magnetron sputtering, because of the high porous surface structure obtained from the sol-gel method^13^. As a result, the surface quality improvement of sol-gel derived ITO due to the use of graphene is higher than that of ITO synthesized by magnetron sputtering due to the use of graphene. However, if ITO with high surface quality obtained from magnetron sputtering is used as previously reported by Liu J. *et al*., the percentage reduction of sheet resistance will be low, since the reduction of sheet resistance through the improvement of surface quality of ITO is limited, and the improvement of electrical properties is negligible, indicating the unique properties of graphene as a hybrid electrode on ITO^22^.

Figure 7(e) shows the I-V characteristics, where the reciprocal gradient of the graph indicates the total resistance between the two contacts, including the contact resistance. The contact resistances of Cu-graphene and Cu-ITO are small, and can be maintained at a similar value by using factors such as the conditions of the Cu deposition method, and the dimensions of Cu contacts^60,61^. It is imperative to identify contact resistance of Cu/graphene, Cu/ITO and Cu/graphene with ITO when comparing sheet resistance of each case since contact resistance may affect variation of sheet resistance. However, contact resistance depends on the size of Cu contact, it is not a good point of comparison. Instead, contact resistivity can be used. Contact resistivity of Cu/graphene, Cu/ITO and Cu/graphene with ITO were obtained as 1.594 × 10^−5^, 1.429 × 10^−5^ and 1.530 × 10^−5^ Ωcm^2^ respectively, by using transmission line measurement (TLM) method (see Supplementary Fig. S2)^62^. Contact resistance values are small and revealed no significant variation in each case, which confirms contact resistance does not significantly affect improvement of the conductivity of graphene/ITO bi-film, compared with individual ITO and graphene. In the present work, the same process conditions and same size Cu contacts were used to deposit Cu contacts, and the distance between two Cu contacts is wide relative to the contact area of Cu, indicating that the information from the I-V curves is mostly from the area between two contacts. Figure 7(e) shows the high gradient of the V-I graph of graphene/ITO bi-film, which implies a lower resistance than those of both the ITO film and the graphene. The optical band gap (E_g_) can be determined by using the plot of (αhν)^2^ versus hν, where α is the absorption coefficient, hν is the incident photon energy, and α can be calculated as follows:^7,63,64^

7 $$\alpha =(\frac{1}{d})\times \,\mathrm{ln}(\frac{1}{T})$$

where, d is the thickness of the film, and T is the optical transmittance. Further, E_g_ is related to α and hν as described in Tauc’s relationship, as follows:^7,16,63,65^

8 $${(\alpha h\nu )}^{2}={\alpha }_{0}(h\nu -{E}_{g})$$

where, ${\alpha }_{0}$ is a constant (the edge width parameter). Figure 7(f) shows the (αhν)^2^ versus hν plot of graphene/ITO bi-film, and the obtained optical band gap of the bi-film was around 3.82 eV. The E_g_ is highly dependent on the carrier concentration, and the wide E_g_ is related to the increment of the concentration of carrier charges, which can be explained by the Moss-Burnstein effect^66^.

### Data availability

All data generated or analysed during this work are included in this article.

## Electronic supplementary material

Supplementary figures and table
