# Supplementary material for: Fabrication of highly conductive graphene/ITO transparent bi-film through CVD and organic additives-free sol-gel techniques
Source: Sci Rep. 2017 Dec 19;7:17868. doi: 10.1038/s41598-017-18063-w (PMC5736725; doi:10.1038/s41598-017-18063-w)
Supplement: Supplementary file 1 — Supplementary figures and table [file 41598_2017_18063_MOESM1_ESM.pdf]

Fabrication of highly conductive graphene/ITO transparent bi-film through CVD and organic additives-free sol-gel techniques

*Bastian Waduge Naveen Harindu Hemasiri, Jae-Kwan Kim, Ji-Myon Lee**

Department of Printed Electronics Engineering, Sunchon National University, Suncheon, Jeonnam 57922, South Korea

* Corresponding author, e-mail: jimlee@sunchon.ac.kr

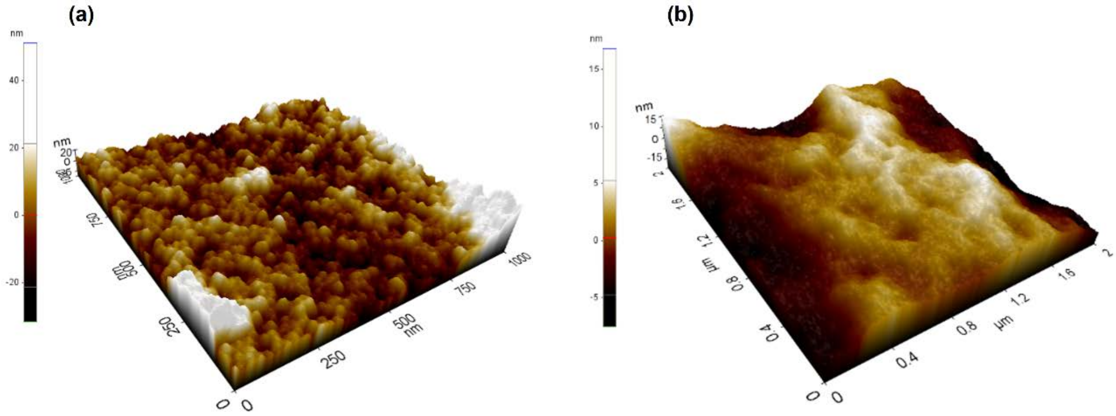

Supplementary Fig. S1: AFM (atomic force microscopy) scan of (a) 6 times spin coated 100 ± 2 nm thick ITO film on plasma treated glass, (b) graphene/ITO bi-film.

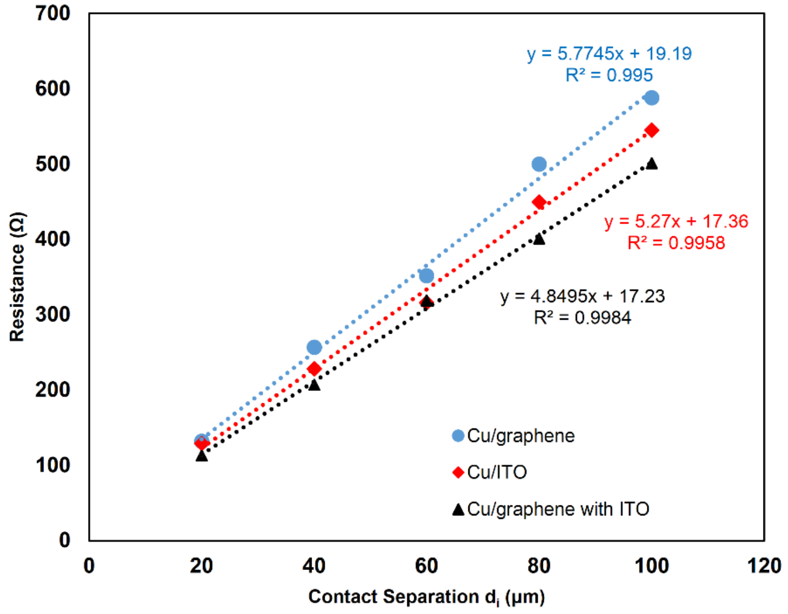

Supplementary Fig. S2: Resistance vs. contact separation graph for Cu/graphene, Cu/ITO and Cu/graphene with ITO.

Supplementary Table S1. The Hall Effect measurement data

Material	Surface carrier concentration ($1/cm^2$)	Carrier mobility (cm^2/Vs)
ITO	1.529E+15	41.375
Graphene	1.538E+14	847.163
Graphene/ITO	1.739E+15	49.015
